# Cannabinoid Signaling in Kidney Disease

**DOI:** 10.3390/cells12101419

**Published:** 2023-05-18

**Authors:** Liana Arceri, Thanh Khoa Nguyen, Shannon Gibson, Sophia Baker, Rebecca A. Wingert

**Affiliations:** Department of Biological Sciences, Center for Stem Cells and Regenerative Medicine, Center for Zebrafish Research, Boler-Parseghian Center for Rare and Neglected Diseases, Warren Center for Drug Discovery, University of Notre Dame, Notre Dame, IN 46556, USA; larceri@nd.edu (L.A.); knguyen8@nd.edu (T.K.N.); sgibson4@nd.edu (S.G.); sbaker9@nd.edu (S.B.)

**Keywords:** kidney, nephron, cannabinoid receptor 1, podocyte, chronic kidney disease, fibrosis, acute kidney injury, cannabinoid receptor 2

## Abstract

Endocannabinoid signaling plays crucial roles in human physiology in the function of multiple systems. The two cannabinoid receptors, CB1 and CB2, are cell membrane proteins that interact with both exogenous and endogenous bioactive lipid ligands, or endocannabinoids. Recent evidence has established that endocannabinoid signaling operates within the human kidney, as well as suggests the important role it plays in multiple renal pathologies. CB1, specifically, has been identified as the more prominent ECS receptor within the kidney, allowing us to place emphasis on this receptor. The activity of CB1 has been repeatedly shown to contribute to both diabetic and non-diabetic chronic kidney disease (CKD). Interestingly, recent reports of acute kidney injury (AKI) have been attributed to synthetic cannabinoid use. Therefore, the exploration of the ECS, its receptors, and its ligands can help provide better insight into new methods of treatment for a range of renal diseases. This review explores the endocannabinoid system, with a focus on its impacts within the healthy and diseased kidney.

## 1. Introduction: Overview of Cannabinoid Signaling

The endocannabinoid system (ECS) is an important signaling pathway that involves the binding of lipid ligands, known as cannabinoids, to cannabinoid receptors, and it encompasses, as well, the metabolic enzymes of endocannabinoids. The signaling pathway consists of two main receptors, cannabinoid receptor 1 (CB1) and cannabinoid receptor 2 (CB2) and more specifically involves endogenous cannabinoid ligands, also known as endocannabinoids [1,2]. The ECS is found throughout multiple body systems and aids in a growing list of cellular functions and processes. Recent studies have highlighted the prominent role the ECS may play in renal health and function. Chronic kidney disease (CKD), including renal fibrosis and diabetic kidney disease, has been linked to cannabinoid signaling, specifically including CB1. CB2 has also been identified within the human kidney; however, recent findings indicate that CB2 activity within the kidney is complicated, contradictory, and not as well-characterized [3]. Thus, although CB2 is emerging as a target of study for renal disease, we will focus primarily on CB1 in this review.

The kidney is a vital player in human physiology and its homeostasis. It is involved in the processing and removal of metabolic byproducts and toxins, regulation of body fluid volumes, maintenance of electrolyte levels, and hormone production [4,5]. Damage to and disease of the kidney can present themselves through either an acute process, such as in acute kidney injury (AKI), or in a chronic process, such as in chronic kidney disease (CKD) [6,7,8]. CKD continues to increase in prevalence worldwide, along with some of its major contributors, such as diabetes, obesity, hypertension, and autoimmune diseases [9]. It is also one of the leading causes of global mortality, posing a great issue, especially in low—and middle—income regions that lack proper treatments and resources [9]. Incidents of AKI are also being reported at an increasing rate, as a result of synthetic drug abuse, creating a new facet to the problem of kidney disease [10].

**Figure 1:**
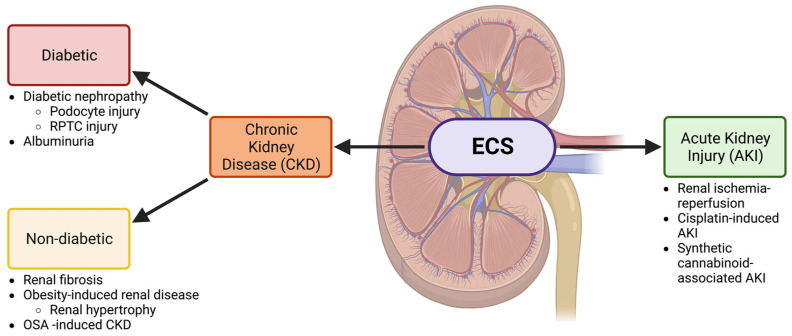
The ECS is involved in both CKD and AKI. A schematic showing the involvement of the ECS in the kidney and the relationship between the nephropathies that will be discussed in this review. Both acute kidney injury (AKI) and chronic kidney disease (CKD) will be investigated. CKD is further divided into diabetic and non-diabetic chronic kidney disease and is associated with several conditions discussed throughout this review. Other abbreviations: renal proximal tubular cell (RPTC) and obstructive sleep apnea (OSA). Created with BioRender.com.

Therefore, understanding the mechanisms that contribute to kidney disease, both chronic and acute, is vital to greater public health efforts. As the endocannabinoid system has recently been gaining attention in the context of kidney disease, it has the potential to unlock new therapies and avenues of study. In this review, we will explore the ECS, with an emphasis on CB1 and its ligands, in the context of renal health (Figure 1). We will then dive deeper into the role the ECS plays in CKD (both diabetic and non-diabetic) and AKI, as well as potential therapies and future directions for a better understanding of this pathway within renal function and health.

### 1.1. The Cannabinoid Receptors: An Emphasis on CB1

CB1 and CB2 are transmembrane G-protein coupled receptors (GPCRs) that aid in multiple cellular functions that influence cell survival and fate [2,11]. GPCRs, more generally, are eukaryotic-specific membrane receptors involved in the translation of external signals into cellular responses. Signals can include light, lipids, sugars, peptides, and proteins [12]. GPCRs are also the largest and most diverse classification of membrane receptors found within eukaryotes [13]. More specifically, cannabinoid receptors are class A GPCRs. Class A GPCRs are the largest subfamily of the GPCR family and are involved in a plethora of cellular signaling processes [12,13]. Such as other class A GPCRs, CB1 and CB2 both have a 7-transmembrane helices domain with a main binding site and an allosteric modulatory binding pocket [12]. Interestingly, recent crystal structures have indicated a complex binding-site network, which has implications for drug-discovery, specifically in regard to the use of phytocannabinoids, such as THC [14].

**Figure 2:**
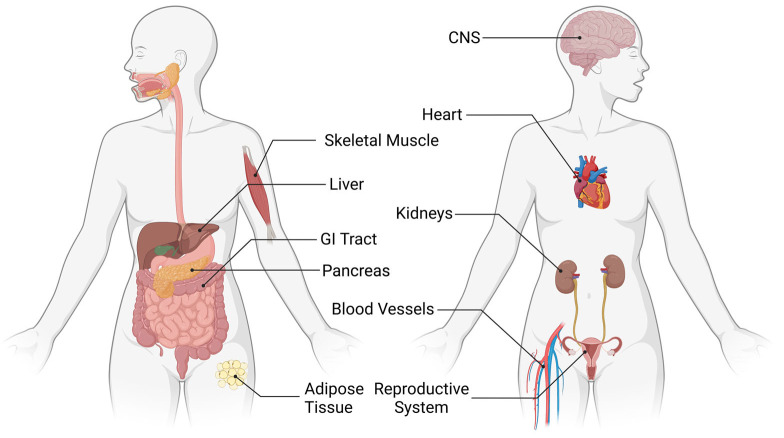
CB1 in the human body. Diagram highlighting the body systems in which CB1 is located and active within the human body. Organs and systems in which CB1 has been studied include the CNS, heart, blood vessels, skeletal muscle, gastrointestinal, liver, pancreas, adipose tissue, renal, and reproductive systems. Created with BioRender.com.

CB1 has been best characterized within the nervous system, as it is one of the most abundant GPCRs in the central nervous system (CNS). More specifically, CB1 is found in high levels within the hippocampus, neocortex, basal ganglia, cerebellum, the respiratory system, and brainstem [2,15]. The endocannabinoid system, more generally within the CNS, is an important regulator of synaptic function and can therefore regulate neural functions, such as cognition, motor control, appetite, pain, and behavior [16]. CB1 has been further studied and characterized within the reproductive tract, skeletal muscular system, gastrointestinal tract, blood vessels, heart, pancreas, adipose tissue, kidney, and liver (Figure 2) [2,17,18,19,20,21].

**Figure 3:**
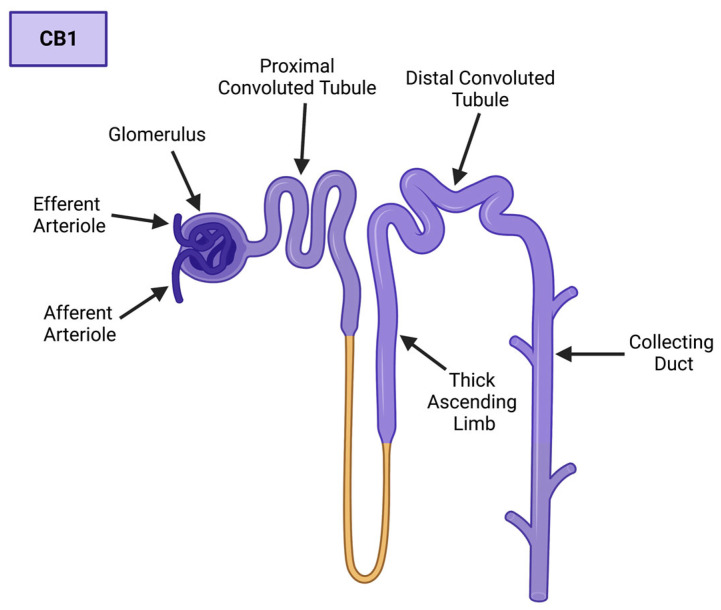
CB1 expression in the human nephron. CB1 is expressed within the glomerulus, efferent and afferent arterioles, proximal and distal convoluted tubules, the thick ascending limb, and the collecting duct. CB1 expression is indicated by purple hue. Created with BioRender.com.

With a focus on the kidney, CB1 has been identified within in the glomeruli, afferent and efferent arterioles, proximal convoluted tubule, thick ascending limb, distal convoluted tubule, and the collecting duct (Figure 3) [22,23,24,25,26,27,28]. Regarding specific renal cell populations, CB1 expression is found within the podocytes, mesangial, endothelial cells, tubular cells, and intercalated cells within their respective regions of the nephron [22,23,24,25,26,27,28]. CB2 has recently been studied in the kidney, specifically in the context of renal fibrosis; however, its role in renal physiology is not as well-characterized, or as prominent, as its counterpart [3,29]. However, CB2 has been well-characterized as a key player in the immune system, as it is found within the thymus, gastrointestinal tract, spleen, bone marrow, and hematopoietic stem cells [2,20,30].

### 1.2. The Endocannabinoids of CB1

**Figure 4:**
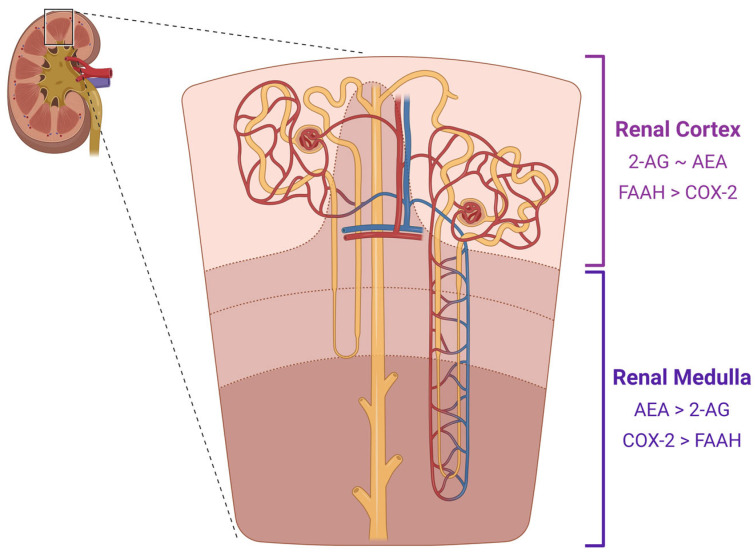
Diagram of CB1 ligands and their degradative enzymes in the kidney. The endocannabinoids, AEA and 2-AG are found in varying levels within the renal cortex and renal medulla. Their degradative enzymes are found in an opposing relative amount within the cortex and medulla as well. Created with BioRender.com.

Endocannabinoids are fatty acid-derived ligands that are produced on command to influence the activity of the cannabinoid receptors [1,31]. Membrane phospholipids are metabolized in response to various stimuli, such as changes in intracellular calcium levels [15]. Anandamide, the first identified endocannabinoid, was identified by Devane et al. in 1992 [32]. However, Mechoulam et al. discovered 2-arachidonoylglycerol (2-AG) not long after in 1995 [33]. AEA is a partial agonist of CB1, and 2-AG is a full agonist that binds to both CB1 and CB2 [1,14,20,34,35]. Further, 2-AG is also recognized as the most abundant endocannabinoid [1,34]. These two ligands are major players in the endocannabinoid signaling pathway within several tissues [35] and, more specifically, have been found in high levels within the kidney. The kidney can be characterized by its higher-than-average level of AEA and its degrading enzyme, compared to other organs within the human body [23,36]. The relative levels of AEA and 2-AG, however, are specific to distinct kidney regions [23,36]. AEA is found in a higher concentration within the renal medulla compared to the cortex [23,36]. On the other hand, 2-AG has similar concentrations in the medulla and cortex and, interestingly, in similar levels to AEA within the cortex (Figure 4) [36].

AEA biosynthesis can be performed through multiple pathways; however, it is initiated by the creation of membrane-bound N-arachidonoyl phosphatidyl ethanolamine (NAPE) [31,37]. The formation of NAPE can be either calcium-dependent or independent [31]. NAPE then converts to create AEA, which can be performed through multiple processes as well [18]. However, the exact mechanism in which NAPE is synthesized within the kidney remains elusive [31]. Interestingly, the AEA-degrading enzymes, such as fatty acid amide hydrolase (FAAH) and cyclooxygenase-2 (COX-2), are found in high levels within the kidney [23,36,37,38,39]. Through western blots and immunostaining analysis performed by Ritter et al. in 2012, FAAH was found in higher levels within the renal cortex, whereas COX-2 was better enriched in the renal medulla (Figure 4) [36]. This opposed distribution of these two enzymes infers a dynamic relationship between the two. FAAH degrades AEA into arachidonic acid and ethanolamine, whereas COX-2 degrades AEA into *N*-ethanolamide analogs of prostaglandins, also known as prostamides [36,40]. Other enzymes responsible for AEA degradation are additionally found within the kidney, such as lipoxygenases and cytochrome P450s [31].

The biosynthesis of 2-AG is similar to that of AEA, as it can be performed through a variety of pathways as well. However, 2-AG synthesis is primarily performed by diacylglycerol lipase (DAGL) with the use of 2-arachidonoyl-phosphatidylinositol 4,5-biphosphate (PIP2) as the main precursor [18,41]. Furthermore, 2-AG is then primarily degraded into arachidonic acid and glycerol, which can be performed by multiple enzymes such as monoacylglycerol lipase (MAGL) and FAAH [41,42]. In the context of the kidney specifically, 2-AG metabolism has not been well-characterized; however, high levels of this endocannabinoid have been reported in cases of acute kidney injury (AKI) [43].

### 1.3. Cellular Processes of the ECS

**Figure 5:**
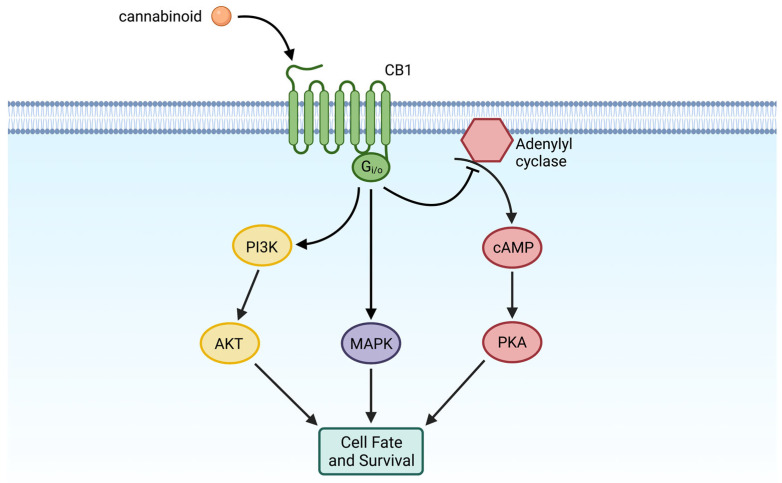
CB1 activity and involvement in cellular pathways. Simplified diagram of cannabinoid receptor 1 (CB1) and some of the cellular pathways in which it plays a role. CB1 is a G-protein coupled receptor that inhibits the activity of adenylyl cyclase and the subsequent cyclic AMP (cAMP) cascade, and it regulates both the mitogen-activated protein kinase (MAPK) and phosphoinositide 3-kinase/protein kinase B (PI3K/AKT) pathways. All three pathways play a role in cell fate and survival. Created with BioRender.com.

The cannabinoid signaling pathway has been shown to be involved in multiple cell-signaling pathways that influence cell survival, differentiation, proliferation, and death [2,11,44]. It has been well-characterized that CB1 inhibits adenylyl cyclase activity, ultimately decreasing cyclic adenosine monophosphate (cAMP) levels [45,46]. This relationship between CB1 and adenylyl cyclase was first introduced, and heavily studied, through the use of mouse spleen cells and Δ9-THC [45,46]. However, there is now evidence that suggests that CB1 activates other important cellular pathways, such as the mitogen-activated protein kinase (MAPK) and the phosphoinositide 3-kinase/protein kinase B (PI3K/Akt) pathways (Figure 5) [11,47,48,49,50]. CB1, being a regulator of these cellular pathways, therefore has the potential to play a role in cell survival and fate. Within the context of the kidney, CB1 activates the MAPK signaling and promotes cisplatin-induced nephropathy, which will be discussed later in this review [50]. Interestingly, the activation of the PI3K/AKT pathway is associated with injury to podocytes, which are crucial for kidney function and health [51], although a formal link between CB1 and podocyte injury via PI3K/Akt activation has yet to be made [22,52,53]. The ECS has been associated with other cellular pathways that are involved in oxidative stress; however, these will not be discussed for the purposes of this review.

## 2. The ECS and Chronic Kidney Disease

### 2.1. Diabetic Kidney Disease

The function of endocannabinoids and their receptors have been linked to diabetic nephropathy, the leading cause of kidney disease in the United States and an initiator of end stage kidney disease [5,54,55]. Diabetic nephropathy can contribute to a decrease in the life expectancies of patients, and there are currently no fully effective treatments. Multiple studies have been aimed to better elucidate the roles of endocannabinoid signaling and its effects on diabetic nephropathy, highlighting the role endocannabinoid signaling plays in podocyte and renal proximal tubular cell (RPTC) physiology (Figure 1) [24,26,53].

Podocytes are highly specialized cells in the kidney that play important roles in glomerular filtration. Interestingly, CB1 has been identified within the podocytes, allowing the ECS to be a target for study [53]. It has been well-characterized that the ECS plays a major role in diabetes and metabolic diseases, which in turn brings focus to diabetic nephropathy [56,57,58,59]. In a 2014 study performed by Jourdan et al., high glucose levels were found to affect CB1 activation in podocytes, causing increased *Cnr1* expression [54]. The overactivation of CB1 resulted in podocyte damage, due to both hyperglycemia and increased renin–angiotensin–aldosterone (RAAS) activity, causing diabetic nephropathy. The potential therapeutic benefits of blocking endocannabinoid signaling in podocytes and its effect on diabetic nephropathy was studied using JD5037, a peripheral CB1 antagonist. Peripheral CB1 antagonism prevented the deterioration of kidney function when applied to prediabetic mice and helped reverse already seen effects in mice exhibiting diabetic nephropathy. Peripheral CB1 antagonism in Zucker diabetic fatty (ZDF) rats, a popular type-2 diabetes model, prevented hyperglycemia, increased kidney weight, elevated plasma creatine, and increased blood urea nitrogen levels and lead to a reduction in GFR. Xanthine oxidase activity was normalized, and the activation of the RAAS was prevented. Peripheral CB1 antagonism also reversed already developed nephropathy in older mice, including polyuria and albuminuria [54]. Overall, this study helped to identify the potential pharmaceutical capabilities of CB1 antagonism in the prevention and treatment of diabetic kidney disease.

In addition to peripheral CB1 antagonism, a novel mouse model with a podocyte-specific deletion of CB1 was developed by Jourdan et al. in 2018 [60]. It has been previously shown that CB1 activation, specifically in the podocytes, contributes to podocyte injury caused by hyperglycemia and increased RAAS activity [54]. A previous in vivo model highlighted that the addition of high levels of glucose in cultured human podocyte cells created a significant increase in CB1 expression and led to podocyte injury [58]. The study performed by Jourdan et al. in 2014 did observe a *Cnr1* knockdown model in the presence of high glucose levels to study CB1 blockade [54]. However, their more recent study used a *Cnr1* deletion model, allowing them to explore the role of CB1 in the development of diabetic nephropathy in vivo. The mice with the podocyte-specific deletion of *Cnr1* were with streptozotocin (STZ) to induce type-1 diabetic nephropathy and chronic hyperglycemia [60]. The knockout of podocyte-specific *Cnr1* protected glomerular and podocyte function, while also shielding proximal tubular function against injury [60]. Knockout of *Cnr1* also contributes to a decrease in oxidative stress and inflammation induced by diabetes as compared to controls [60]. Overall, CB1 has been identified as being a mechanistic component of podocyte injury specifically by influencing oxidative stress [53,60].

**Figure 6:**
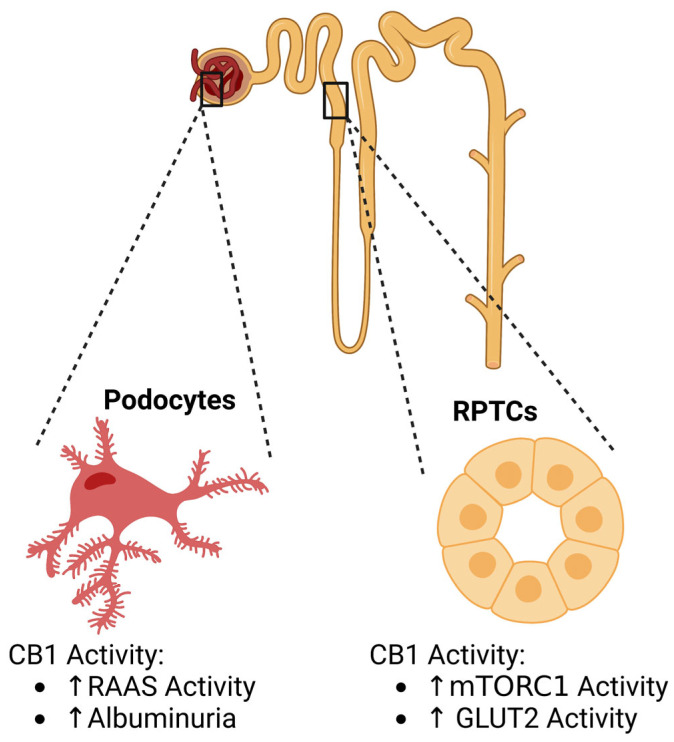
CB1 activity and associations in DKD. Endocannabinoid activity plays roles in podocytes and renal proximal tubule cell (RPTC) physiology. Created with BioRender.com.

A specific characteristic of diabetic nephropathy is albuminuria, which results from the dysregulation of the glomerular filtration barrier (Figure 6) [61]. CB1 has previously been shown to influence albuminuria, as CB1 blockade was found to ameliorate albuminuria [57]. In 2018, Barutta et al. investigated how AM6545, a CB1 receptor antagonist, acted in conjunction with the treatment of perindopril, an ACE-inhibitor, for type 1 diabetic mice [59]. The goal was to see if the connection between ECS and RAS can be applied to a more effective therapy for albuminuria than ACE-inhibitors alone [59]. Once diabetes was established in their mouse models, albuminuria levels were observed with the inclusion of AM6545, perindopril, or the dual therapy of both. Both the AM6545 and perindopril treatments reduced albuminuria by 50%. However, the dual therapy resulted in an albuminuria level comparable to that of the non-diabetic mice, indicating a rescue early diabetic nephropathy. Additionally, a similar trend in podocyte development was observed in the reduction of nephrin and podocin. The single treatments of perindopril or AM6545 helped to rescue nephrin and podocin reduction in immunostaining; however, dual therapy fully rescued the reduction in diabetic mice. The researchers then looked at whether CB1 receptor interferes with retinoic acid (RA) signaling and found that exposure to RA resulted in an increase in nephrin mRNA level. However, treatment with a CB1 receptor agonist, ACEA, reverted this trend by lowering nephrin expression below the control. Additionally, the rescue of other factors such as monocyte infiltration, macrophage polarization towards M2, or an overabundant expression of inflammatory factors were observed in the dual therapy. This study highlights the therapeutic potential of dual therapies by inhibiting both CB1 and RAS to reverse nephropathy in diabetic mice.

Although widely accepted as a glomerular disease, recent studies suggest RPTCs play a role in diabetic kidney disease (DKD) when they are exposed to dysregulated nutrient and energy sensing in diseases such as diabetes and obesity [62,63]. Specifically, the activation of mTORC1, due to a nutrient overload, in the RPTCs was identified as a key driver of diabetic nephropathy [62]. CB1 has been characterized as being expressed within the proximal tubular cells of the human adult kidney and plays roles in RPTC function [24,26,39,64,65]. Interestingly, endocannabinoid signaling has been associated with proximal tubular hypertrophy, which is associated with diabetic nephropathy [64]. As shown by Hinden et al. in 2022 [63], endocannabinoid signaling plays a key role in regulating mTORC1 signaling in RPTCs in normal and disease states. Hyperglycemia, a known contributing factor of diabetic nephropathy was shown to stimulate endocannabinoid signaling and the subsequent activation of CB1. Therefore, the relationship between CB1 and mTORC1 was studied. CB1-deficient diabetic mice demonstrated a decrease in mTORC1 signaling and levels of GLUT2, preserving kidney function, inferring that CB1 activity stimulates mTORC1 activity, leading to DKD advancement in mice. However, non-diabetic mice that do not contain CB1 show increased mTORC1 signaling, due to increased amino acid transport [63]. CB1 activity has also been found to mediate obesity-induced renal lipotoxicity by downregulating AMPK signaling and increasing lipid accumulation in the RPTCs. CB1 antagonists may offer therapeutic benefits by preventing GLUT2 translocation to the apical membrane, causing enhanced glucose uptake and increasing AMPK phosphorylation [63]. Taken together, these studies have revealed crucial physiological changes associated with CB1 in podocytes and RPTCs in diabetes (Figure 6).

### 2.2. The ECS in Renal Fibrosis and Other Non-Diabetic CKDs

**Figure 7:**
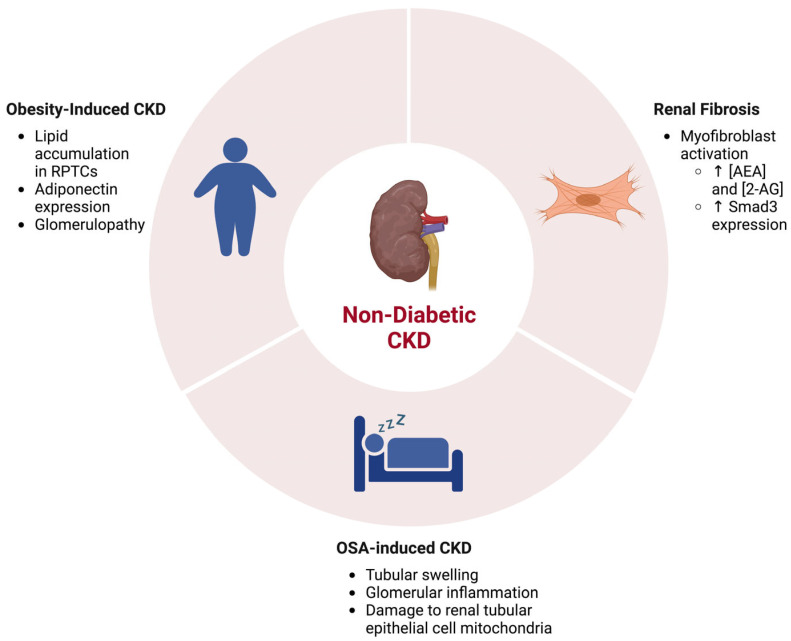
The ECS and associations in NDCKD. Kidney diseases including renal fibrosis, obesity-induced CKD, and obstructive sleep apnea (OSA)-induced CKD are associated with activity alterations in the ECS. Created with BioRender.com.

Chronic kidney disease can also be independent of diabetes. Kidney diseases such as fibrosis and proteinuria can be hereditary or caused by other issues that precede diabetes, such as pregnancy [66,67]. Interestingly, the endocannabinoid system has been linked to non-diabetic chronic kidney diseases (NDCKD) as well. Renal fibrosis is caused by the excessive accumulation of an extracellular matrix, and while it may additionally be a result of DKD, it can additionally originate from non-diabetic chronic injury. Interestingly, CB1 has recently emerged as a key player in its pathogenesis (Figure 7) [67]. In 2015, Lecru et al. aimed to better understand the underlying mechanisms of renal fibrosis [29]. In order to do so, they performed a microarray analysis to compare the gene expressions of fibrotic and normal kidneys. Diseased kidneys were studied using the unilateral ureteral obstruction (UUO) experimental model in mice to induce fibrosis. Surprisingly, the Cnr1 gene, the gene responsible for encoding CB1, was one of the most upregulated genes within the UUO model. This novel finding then lead to the exploration of CB1 expression within renal fibrosis and other diseases. The immunostaining of multiple renal biopsies revealed that CB1 expression increased in renal fibrosis, acute interstitial nephritis, IgA nephropathy, and diabetic nephropathy, indicating a relationship between CB1 expression and kidney function. When further studied in the context of renal fibrosis, CB1 expression was found to be increased in myofibroblasts, the main contributor of fibrosis. In the UUO model, global endocannabinoid levels, including AEA and 2-AG, increased, indicating a change in the overall endocannabinoid signaling system within the context of renal fibrosis. To further validate their findings, a genetic knockout (Cnr1^−/−^) and pharmacological blockade model were used and showed a decrease in fibrosis. Through these CB1 deficiency models, this study determined that CB1 is involved in the development of renal fibrosis [29].

More recent evidence suggests that CB1 agonism may be influencing renal fibrosis through Smad3 signaling [68]. Smad3 expression is a major mediator of renal fibrosis [69,70]. TGF-β1, a mediator of renal fibrosis, activates Smad3, which in turn regulates target genes for renal fibrosis [29,70,71]. More specifically, aspects of fibrosis such as collogen synthesis and the epithelial–mesenchymal transition are dependent on TGF-β1/Smad3 signaling [70]. The results from the Lecru et al. study [29] suggest that CB1 activity acts downstream of TGF-β1. Interestingly, CB1 is hypothesized to be a negative mediator of TGF-β1/Smad3 signaling in pulmonary fibrosis [72]. The study performed by Golosova et al. [68] highlighted that high doses of AEA led to an increase in Smad3 expression, however surprisingly did not affect TGF-β1 expression. Previous studies have indicated that CB1 antagonists, such as AM-251, acted upstream of Smad3 and p38 MAPK in EMT; however, it did not act through CB1 [73]. This finding indicates that CB1 may not play a role in EMT regarding renal fibrosis. It was also hypothesized that CB1 has the potential to act through an alternative pattern, independent of TGF-β1 [68]. Overall, these findings indicate a complicated role for CB1 signaling in renal fibrosis that needs further exploration.

It has been well characterized that the over-activation of the ECS is associated with obesity and obesity-related issues (Figure 7) [74,75]. As a result, the potential relationship between the ECS and obesity-induced CKD is of interest. A previous study treated obese Zucker rat models with CB1 antagonists, which led to the attenuation of proteinuria and improved creatinine clearance, as well as a reduction in renal hypertrophy [75]. This connection between renal injury and CB1 activity highlights the role ECS plays in obesity-induced CKD. While less is known about it than obesity-associated glomerulopathy, renal tubule injury is another facet of obesity-induced CKD that is a potential therapeutic target [76]. A study, performed by Udi et al. in 2017 [65], aimed to focus on RPTCs within the context of obesity-induced CKD, as the RPTCs are particularly sensitive to lipid accumulation. It has been previously shown that the ECS is a key player in obesity; it has been shown to be involved in RPTC function as well, highlighting a possible relationship between endocannabinoid signaling and obesity-related renal pathologies [64,74,75]. Using RPTC-specific CB1-null mice, CB1 deletion was determined to reduce obesity-induced lipid accumulation in the kidney, inferring CB1 plays a role in the development of renal lipotoxicity. This result was then associated with the regulation of the LKB1/AMPK/ACC signaling pathway by the CB1R-coupled Gai/o-PKA axis [65]. Further studies indicated that CB1 helps to regulate the expression of adiponectin, an enzyme involved in glucose regulation and fatty acid degradation [77]. Adiponectin is also regulated by induced NOS (iNOS), another pathway involved in renal dysfunction, which allowed researchers to explore the connection between CB1 signaling and iNOS activity. The association of these two signaling pathways led to the establishment of a CB1/iNOS hybrid antagonist as a possible treatment for obesity-induced CKD [77].

Another NDCKD that has been studied in the context of the ECS is obstructive sleep apnea (OSA)-induced chronic kidney disease (Figure 7). The renal issues associated with OSA are mainly caused by intermittent hypoxia, more specifically [78]. Chronic intermittent hypoxia (CIH) has previously been shown to activate CB1 within bone, triggering metabolic bone disorders, indicating a relationship between CB1 and CIH [79]. It was hypothesized that this disease is due to mitochondrial dysfunctions that cause organ damage and might be able to be regulated by endocannabinoids. CIH is one of the main symptoms of OSA and affects kidney mitochondria. Therefore, patients with untreated OSA can result in CKD [80]. CB1 has been shown to affect mitochondrial function in kidneys and is shown to be very active in human kidney disease [24]. Zhao et al. in 2021 [81] therefore looked at three mitochondrial regulators as well as CB1 expression in the OSA-CIH rat model after the treatment of the CB1 antagonist, rimonabant (Ri). Through the analysis of renal tissue under electron microscopy (EM) and western blot analysis, no morphological abnormalities were found in the control group; however, the CIH group had swelling and a narrow tubule lumen. Tubular epithelial cells were also damaged, and a slightly inflammation was seen within the glomeruli. With the rimonabant treatment, swelling and tubular damage decreased. The CIH group indicated severe structural damage in renal tubular epithelial cell mitochondria, as well as the presence of mitochondria fragments. After rimonabant treatment, the damage to the mitochondria, as well as the presence of mitochondrial fragments, diminished, suggesting that the antagonist helped combat some of the structural damage in the renal mitochondria due to the CIH. Western blotting showed that CB1 expression in the renal tissues of the CIH group was increased, and after treatment with the antagonist, CB1 levels decreased, subsequently decreasing renal injury. Overall, it was concluded that CIH caused by OSA could induce endocannabinoid signaling, which then correlates to renal disease.

## 3. The ECS and Acute Kidney Injury

Renal ischemia-reperfusion (IR) has been known to contribute to AKI development. IR is caused by the impairment and rescue of blood flow to the kidney, commonly associated with injuries associated with partial nephrectomy (PN), transplant, infarction, and sepsis [82,83]. The endocannabinoid system (ECS) has been studied in its relationship with the IR across organs [84]. In the case of IR, the involvement of ECS has been observed in rodent models. For example, it has been observed that renal clamping causes changes in the levels of AEA [85] or 2-AG [43,86]. Additionally, the expression of CB1 and CB2 have also been found to be changed [85,87]. In Rothner et al., 2023 [88], the researchers studied the systemic endocannabinoid (eCB) levels caused by surgical renal IR. The study looked at 16 patients undergoing on-clamp PN and studied several levels of several factors important to the ECS system before renal ischemia, after 10 min of ischemia, and after 10 min from blood reperfusion. The study found that levels of 2-AG and kidney dysfunction biomarkers positively correlated with each other. The study showed that the levels of BUN, glucose, and sCr were elevated after reperfusion. The study did not detect significant changes in the CBs, CB-like molecules, or arachidonic acid (AA) in all patients. The study also showed that there was a significant increase in *N*-acylethanolamines in non-obese patients, however not in obese patients who already had higher levels of *N*-acylethanolamines.

Another issue that currently involves AKI is the inclusion of cisplatin-induced AKI (CIAKI). Cisplatin is a well-established chemotherapeutic agent that can result in AKI in its patients [89,90,91]. This chemotherapeutic drug induces tumor necrosis factor-α (TNF-α), which in turn stimulates cytokine and chemokine expression within the kidney to cause injury [92]. Although this drug is widely used to treat a plethora of cancers, a treatment to reduce the incidences of AKI under its use remains unknown. A study by Mukhopadhyay et al., 2010 [50], aimed to begin exploring a possible connection between CIAKI and the role CB1 plays in oxidative stress and inflammation. In this study, the researchers investigated the effect of pharmacological and genetic inhibition of CB1 receptors on a murine nephropathy model. First, they detected AEA and 2-AG in the mouse kidney and found that cisplatin increased the tissue levels of AEA. Additionally, it was found that with the treatment of AM-281 and SR141716, two CB1 antagonists, renal dysfunction was attenuated. A similar trend was additionally observed in CB1^−/−^ mice. In addition, the researchers looked at histopathological damages and cell death and found that both the pharmacological and genetic inhibition of CB1 helped attenuate the renal damage, as well as reducing cell death in murine kidneys. The researchers further investigated additional factors of stress, such as p38 and JNK MAPK activation or interrelated oxidative/nitrosative stress and found that both the genetic deletion and pharmacological inhibition of CB1 helped mitigate these markers of renal dysfunction. Taken together, these results suggested that CB1 inhibitors could serve as a powerful pharmacotherapy treatment to reduce the nephrotoxicity effects of cisplatin.

Synthetic cannabinoids (SCs) are increasingly known as an abusive drug in young adults. Initially developed in the 1960s for studies on the pharmacology of cannabinoid receptors, SCs are non-polar agonists of the two endocannabinoid receptors, CB1 and CB2 [93,94]. However, not much has been known about their specific pharmacology [95]. SCs have recently become a popular type of drug abuse in young adults, particularly due to the similarities in their effects to cannabis [96]. Additionally, SCs are easily accessible with a low cost and distributed under names such as “Spice” or “K2”. In addition, they are easily consumed, either by smoking, insufflating, or ingesting. The most common renal pathology for renal problems due to SCs are acute kidney injury (AKI), accounting for less than 1% of all cases [97,98,99]. SCs have been known for their ability to reduce inotropic effects, leading to a blood flow reduction to the kidney. SC use can also be associated with cannabinoid hyperemesis syndrome, a condition observed in long-term users who experience symptoms such as vomiting, abdominal discomfort, or nausea [100].

In a case study presented by Acharya et al., 2023 [101], a 16-year-old male was admitted to the Pediatric Department at the University of Florida with symptoms of right flank pain, loss of appetite, constipation, and vomiting. He had no family history of renal disease/disorder; however, he did admit to smoking cannabis and SCs for approximately a year prior to this event and as recently as 2 days before admission. A biopsy of his kidney indicated evidence of severe acute interstitial nephritis. The biopsy showed interstitial inflammation at the cortico–medullary junction and the presence of many eosinophils. This study suggests that the mechanism in which AIN is manifested in the case of SC-use is through antigen processing and presentation and the activation of T-cells.

However, due to their illegal nature, it is difficult to determine the exact constituents of SCs, and therefore, it is difficult to determine the exact cause of AIN. In a previous 2013 case report by Kazory and Aiyer [102], the renal biopsy of a 22-year-old man who used SC regularly revealed acute tubular necrosis with flattened epithelium and focal tubular atrophy. Additionally, immunofluorescence staining revealed the loss of casts, vacuolization, mitosis, and brush border and found the presence of unclear vessels. This study proposed that the direct effect of SC may cause toxic tubular injury to the kidneys of these patients. The study also observed three notable points. First, the reported cases are predominantly male (20 out of 21 cases), with the most common symptoms being nausea, vomiting, and abdominal pain. Second, the most common finding in a renal biopsy is tubular injury (10 out of 12 cases). Third, 25% of patients needed replacement therapy (5 out of 21). The study concluded that SC abuse can result in renal complications that lead to SC-associated AKI.

In addition, several other reports have recorded the nephrotoxicity of SCs. In D’Errico et al., 2022, a broad literature survey was conducted from 21 studies, 14 case reports, and 7 case series, in which they found a broad range of symptoms of SC consumption. Histological findings revealed several kidney dysfunctions, such as acute tubular injury, acute tubulointerstitial nephritis, acute interstitial nephritis, acute tubular necrosis with peritubular medullary capillaritis, and acute glomerular disease [103]. For example, four cases of AKI, linked to SC abuse in Alabama, experienced symptoms such as vomiting, abdominal pain, and nausea after SC usage, and three developed severe AKI, for which their renal biopsy showed acute tubular necrosis (ATN) [104]. However, all patients recovered with appropriate care without the need for a kidney replacement. The Center for Disease Control and Prevention (CDC) also reported multiple cases of AKI as an unanticipated complication from SC abuse, with 16 patients hospitalized in 2012 alone. Biopsies revealed several findings, such as ATN, acute interstitial nephritis, and corticosteroids [105]. While AKI is not a common manifestation of SC abuse, with an approximately 4% incidence rate in a study of 277 cases in 2016, it does present a risk factor for the development of CKD [101,106].

## 4. Therapeutic Potential

As seen thus far, ECS is involved in multiple renal pathologies. As a result, it is being highlighted as a possible therapeutic target. Diseases, such as renal fibrosis and diabetic kidney disease, are worsened by the activation of CB1. Therefore, CB1 antagonists are being explored as therapies for these pathologies. Many studies have investigated both chemical inhibition and genetic manipulation; however, chemical inhibition specifically has pharmaceutical potential. Rimonabant, a well-known CB1 antagonist has been shown to attenuate renal diseases such as CIH-induced kidney disease and renal fibrosis [29,81]. This antagonist was sold on the market in the past, as it was used to achieve weight-loss and was being tested as a cardiovascular drug [107,108]. However, due to the inhibitory effects of the drug in the CNS, rimonabant began to show psychiatric side effects of anxiety and depression [109,110]. Rimonabant was shown to have higher blood–brain barrier penetrance, causing it to have CNS-related side effects [111,112]. As a result, efforts have been made to create CB1 antagonists that possess the ability to inhibit CB1 activity without penetrating the blood–brain barrier. Second and third generation antagonists were thus developed.

Second-generation antagonists have been developed over the past decade and are peripherally restricted [113]. This suggests that these antagonists affect peripheral CB1 receptors and not the CNS. Multiple second-generation CB1 antagonists have already been and are currently being developed [113]. Many second-generation antagonists are being formulated to treat metabolic issues such as obesity, diabetes, etc., without impairing CNS function [113,114,115]. The most notable antagonists used within the context of kidney disease, more specifically, are AM6545 and JD5037. AM6545 has previously been shown to possess the same therapeutic benefits as rimonabant, without CNS-related adverse effects [116,117]. This antagonist was also proven to help treat TGFβ1-mediated renal inflammation and fibrosis, as well as albuminuria and nephrin loss in diabetic kidney disease [117,118]. JD5037 is another peripherally restricted CB1 antagonist that has therapeutic potential within the liver and pancreas [119,120]. In kidney disease, JD5037 has been shown to improve diabetic nephropathy, by improving podocyte function and led to the production of a hybrid/third-generation CB1 antagonist, MRI-1867, which has been shown to improve obesity-induced CKD [54,77].

Third-generation antagonists are also peripherally restricted; however, these antagonists are aiming to be dual-target antagonists. As mentioned previously, in the Udi et al., 2020, study [77], a CB1/iNOS antagonist was developed to treat obesity-induced CKD. Prior to this hybrid antagonist, iNOS inhibitors lacked oral bioavailability, and there was no effective therapy for fibrosis approved for use [77]. In 2016, Cinar et al. developed MRI-1867, a hybrid CB1/iNOS antagonist that was shown to alleviate liver fibrosis [121]. In their study, Udi et al. [77] showed that not only was MRI-1867 effective in treating obesity-induced CKD, it was more effective than the monotherapy of a peripherally restricted CB1 antagonist, JD5037, as it simultaneously inhibited two major mediators. This drug therefore highlights the beneficial potential of formulating CB1 antagonists that have additional targets, such as AMPK [113]. Interestingly, a recent study examined the effects of JD5037 in combination with metformin, an AMPK activator, in pulmonary hypertension and discovered that the conjunction of these two drugs together proved to be more effective than the monotherapies, highlighting the potential for a CB1/AMPK dual-target drug [122]. A very current issue that has the potential to benefit from a CB1/iNOS hybrid antagonist is the development of the long-term effects of COVID-19, or long “COVID” [123]. Acute kidney injury, which is influenced by both CB1 and iNOS activity, is a common complication of COVID-19. The development of a single medication that can inhibit both CB1 and iNOS activity can be a desirable therapy for COVID-19 and the accompanying AKI, as well as other CB1 and iNOS-associated complications such as lung injury [123].

Other exogenous cannabinoids have also been known to treat renal disease, such as nephrotoxicity-induced symptoms due to cisplatin. Studies have shown that cannabinoids can exert anti-inflammatory properties via the CB1 and CB2 in the immune cells and central nervous system, yet there existed certain limitations, such as the development of psychoactive effects [124,125], and some recent reviews have comprehensively covered the role of CB2 in immune cells [126,127,128]. In recent years, cannabidiol (CBD) has emerged as a powerful alternative, as its low affinity for CB1 and CB2 receptors does not cause psychoactive effects. However, it has also been known to exert antioxidant, immunomodulatory, and anti-inflammatory effects [129,130]. CBD has been recognized as acting as a CB1 antagonist; however, recent evidence suggests CBD may be a negative allosteric modulator [131]. In Pan et al., 2009, researchers studied the effects of CBD using a cisplatin-induced nephropathy mouse model [132]. They found that CBD helped attenuate many renal dysfunctions from cisplatin-induced nephrotoxicity in mice. For example, dose-dependent treatment with CBD reduced levels of Creatinine and BUN level in mice administered with cisplatin. Additionally, histological examination showed a reduction in the renal tubular damage in cisplatin-treated mice. A TUNEL assay marking apoptotic cells also showed a marked decrease in CBD treatment. CBD was additionally found to reduce cisplatin-induced inflammation, marked by an attenuated level of both TNF-α and IL1β compared to cisplatin-administered mice alone. They additionally found that CBD reduced ROS formation and caused an increased expression of RENOX (NOX4) and NOX1, two superoxide-generating enzymes.

## 5. Conclusions

Recent studies have identified CB1 as a potential target of exploration regarding kidney health and function. As highlighted in this review, the activity of CB1 and its endogenous ligands have been identified as key players in multiple renal pathologies, including chronic and acute diseases. From this review, we can appreciate the very vast role endocannabinoid signaling plays in nephropathies. CB1 activity has been characterized as a mediator of renal fibrosis through the activation of myofibroblasts. Diabetic kidney diseases, involving changes in both the glomerular and proximal tubular cell populations, are associated with the activation of CB1 [133]. Renal ischemia-reperfusion has additionally been shown to be associated with changes in endocannabinoid levels, introducing another facet of ECS involvement. However, we can also highlight that by the inhibition of CB1 we can expand possible therapies for nephropathies, such as cisplatin-induced AKI, amongst other adverse diseases. Interestingly, several studies show that CB2 activation can additionally be protective in several kidney diseases [134,135,136,137,138,139,140,141,142]. It is also increasingly important to recognize the role cannabinoid signaling plays in kidney disease, as well as overall health, as synthetic cannabinoids continue to exist out on the market, introducing a new public health threat. Another facet to the field of cannabinoid research are the limitations of applying insights from animal models to humans. For example, animal models of DKD do differ from the human system, and clinical data taken from patients and pharmacological agents that target CB receptors may have very different effects, including side effects such as the adverse psychotic effects. Overall, the research aiming to investigate the ECS, specifically in the context of the kidney, is opening new avenues of study and discussion to help us better our understanding of renal function.

## Data Availability

Not applicable.

## References

[1] Fezza F., Bari M., Florio R., Talamonti E., Feole M., Maccarrone M. (2014). Endocannabinoids, related compounds and their metabolic routes. Molecules.

[2] Howlett A.C., Barth F., Bonner T.I., Cabral G., Casellas P., Devane W.A., Felder C.C., Herkenham M., Mackie K., Martin B.R. (2002). International union of pharmacology. XXVII. classification of cannabinoid receptors. Pharmacol. Rev..

[3] Zhou L., Zhou S., Yang P., Tian Y., Feng Z., Xie X., Liu Y. (2018). Targeted inhibition of the type 2 cannabinoid receptor is a novel approach to reduce renal fibrosis. Kidney Int..

[4] Chambers B.E., Weaver N.E., Wingert R.A. (2023). The “3Ds” of growing kidney organoids: Advances in nephron development, disease modeling, and drug screening. Cells.

[5] Chua J.T., Argueta D.A., DiPatrizio N.V., Kovesdy C.P., Vaziri N.D., Kalantar-Zadeh K., Moradi H. (2019). Endocannabinoid system and the kidneys: From renal physiology to injury and disease. Cannabis Cannabinoid Res..

[6] McCampbell K.K., Wingert R.A. (2012). Renal stem cells: Fact or science fiction?. Biochem. J..

[7] Li Y., Wingert R.A. (2013). Regenerative medicine for the kidney: Stem cell prospects & challenges. Clin. Transl. Med..

[8] McKee R.A., Wingert R.A. (2015). Zebrafish renal pathology: Emerging models of acute kidney injury. Curr. Pathobiol. Rep..

[9] Kovesdy C.P. (2022). Epidemiology of chronic kidney disease: An update 2022. Kidney Int. Suppl..

[10] Pendergraft W.F., Herlitz L.C., Thornley-Brown D., Rosner M., Niles J.L. (2014). Nephrotoxic effects of common and emerging drugs of abuse. Clin. J. Am. Soc. Nephrol..

[11] Peyravian N., Deo S., Daunert S., Jimenez J.J. (2020). Cannabidiol as a novel therapeutic for immune modulation. Immunotargets Ther..

[12] Shahbazi F., Grandi V., Banerjee A., Trant J.F. (2020). Cannabinoids and cannabinoid receptors: The story so far. iScience.

[13] Zhou Q., Yang D., Wu M., Guo Y., Guo W., Zhong L., Cai X., Dai A., Jang W., Shakhnovich E.I. (2019). Common activation mechanism of class A GPCRs. eLife.

[14] Hua T., Vemuri K., Pu M., Qu L., Han G.W., Wu Y., Zhao S., Shui W., Li S., Korde A. (2016). Crystal structure of the human cannabinoid receptor CB_1_. Cell.

[15] Kendall D.A., Yudowski G.A. (2017). Cannabinoid receptors in the central nervous system: Their signaling and roles in disease. Front. Cell. Neurosci..

[16] Castillo P.E., Younts T.J., Chávez A.E., Hashimotodani Y. (2012). Endocannabinoid signaling and synaptic function. Neuron.

[17] Allam S., Paris E., Lazcano I., Bitterman P., Basu S., O’Donnell J., Barua A. (2022). Detection of cannabinoid receptor expression by endometriotic lesions in women with endometriosis as an alternative to opioid-based pain medication. J. Immunol. Res..

[18] Dao M., François H. (2021). Cannabinoid receptor 1 inhibition in chronic kidney disease: A new therapeutic toolbox. Front. Endocrinol..

[19] Hasenoehrl C., Taschler U., Storr M., Schicho R. (2016). The gastrointestinal tract—A central organ of cannabinoid signaling in health and disease. Neurogastroenterol. Motil..

[20] Haspula D., Clark M.A. (2020). Cannabinoid receptors: An update on cell signaling, pathophysiological roles and therapeutic opportunities in neurological, cardiovascular, and inflammatory diseases. Int. J. Mol. Sci..

[21] Liu L.Y., Alexa K., Cortes M., Schatzman-Bone S., Kim A.J., Mukhopadhyay B., Cinar R., Kunos G., North T.E., Goessling W. (2016). Cannabinoid receptor signaling regulates liver development and metabolism. Development.

[22] Barutta F., Bellini S., Gruden G. (2022). Mechanisms of podocyte injury and implications for diabetic nephropathy. Clin. Sci..

[23] Deutsch D.G., Goligorsky M.S., Schmid P.C., Krebsbach R.J., Schmid H.H., Das S.K., Dey S.K., Arreaza G., Thorup C., Stefano G. (1997). Production and physiological actions of anandamide in the vasculature of the rat kidney. J. Clin. Investig..

[24] Drori A., Permyakova A., Hadar R., Udi S., Nemirovski A., Tam J. (2019). Cannabinoid-1 receptor regulates mitochondrial dynamics and function in renal proximal tubular cells. Diabetes Obes. Metab..

[25] Koura Y., Ichihara A., Tada Y., Kaneshiro Y., Okada H., Temm C.J., Hayashi M., Saruta T. (2004). Anandamide decreases glomerular filtration rate through predominant vasodilation of efferent arterioles in rat kidneys. J. Am. Soc. Nephrol..

[26] Larrinaga G., Varona A., Pérez I., Sanz B., Ugalde A., Cándenas M.L., Pinto F.M., Gil J., López J.I. (2010). Expression of cannabinoid receptors in human kidney. Histol. Histopathol..

[27] Lin C., Hsu Y., Lee P., Lei C., Wang J., Huang Y., Wang S.Y., Wang F. (2014). Cannabinoid receptor 1 disturbance of PPARγ2 augments hyperglycemia induction of mesangial inflammation and fibrosis in renal glomeruli. J. Mol. Med..

[28] Silva G.B., Atchison D.K., Juncos L.I., García N.H. (2013). Anandamide inhibits transport-related oxygen consumption in the loop of henle by activating CB1 receptors. Am. J. Physiol. Renal Physiol..

[29] Lecru L., Desterke C., Grassin-Delyle S., Chatziantoniou C., Vandermeersch S., Devocelle A., Vernochet A., Ivanovski N., Ledent C., Ferlicot S. (2015). Cannabinoid receptor 1 is a major mediator of renal fibrosis. Kidney Int..

[30] Esain V., Kwan W., Carroll K.J., Cortes M., Liu S.Y., Frechette G.M., Sheward L.M.V., Nissim S., Goessling W., North T.E. (2015). Cannabinoid receptor-2 regulates embryonic hematopoietic stem cell development via prostaglandin E2 and P-selectin activity. Stem Cells.

[31] Ritter J.K., Li G., Xia M., Boini K. (2016). Anandamide and its metabolites: What are their roles in the kidney?. Front. Biosci. (Schol. Ed.).

[32] Devane W.A., Hanus L., Breuer A., Pertwee R.G., Stevenson L.A., Griffin G., Gibson D., Mandelbaum A., Etinger A., Mechoulam R. (1992). Isolation and structure of a brain constituent that binds to the cannabinoid receptor. Science.

[33] Mechoulam R., Ben-Shabat S., Hanus L., Ligumsky M., Kaminski N.E., Schatz A.R., Gopher A., Almog S., Martin B.R., Compton D.R. (1995). Identification of an endogenous 2-monoglyceride, present in canine gut, that binds to cannabinoid receptors. Biochem. Pharmacol..

[34] Scherma M., Masia P., Satta V., Fratta W., Fadda P., Tanda G. (2019). Brain activity of anandamide: A rewarding bliss?. Acta Pharmacol. Sin..

[35] Sugiura T., Kobayashi Y., Oka S., Waku K. (2002). Biosynthesis and degradation of anandamide and 2-arachidonoylglycerol and their possible physiological significance. Prostaglandins Leukot. Essent. Fatty Acids.

[36] Ritter J.K., Li C., Xia M., Poklis J.L., Lichtman A.H., Abdullah R.A., Dewey W.L., Li P. (2012). Production and actions of the anandamide metabolite prostamide E2 in the renal medulla. J. Pharmacol. Exp. Ther..

[37] Sugiura T., Waku K. (2002). Cannabinoid receptors and their endogenous ligands. J. Biochem..

[38] Long J.Z., LaCava M., Jin X., Cravatt B.F. (2011). An anatomical and temporal portrait of physiological substrates for fatty acid amide hydrolase. J. Lipid Res..

[39] Sampaio L.S., Taveira Da Silva R., Lima D., Sampaio C.L.C., Iannotti F.A., Mazzarella E., Di Marzo V., Vieyra A., Reis R.A.M., Einicker-Lamas M. (2015). The endocannabinoid system in renal cells: Regulation of Na(+) transport by CB1 receptors through distinct cell signalling pathways. Br. J. Pharmacol..

[40] Yu M., Ives D., Ramesha C.S. (1997). Synthesis of prostaglandin E2 ethanolamide from anandamide by cyclooxygenase-2. J. Biol. Chem..

[41] Tsuboi K., Uyama T., Okamoto Y., Ueda N. (2018). Endocannabinoids and related N-acylethanolamines: Biological activities and metabolism. Inflamm. Regen..

[42] Lemtiri-Chlieh F., Levine E.S. (2022). 2-AG and anandamide enhance hippocampal long-term potentiation via suppression of inhibition. Front. Cell. Neurosci..

[43] Moradi H., Oveisi F., Khanifar E., Moreno-Sanz G., Vaziri N.D., Piomelli D. (2016). Increased renal 2-arachidonoylglycerol level is associated with improved renal function in a mouse model of acute kidney injury. Cannabis Cannabinoid Res..

[44] Guzmán M. (2003). Cannabinoids: Potential anticancer agents. Nat. Rev. Cancer.

[45] Kaminski N.E., Koh W.S., Yang K.H., Lee M., Kessler F.K. (1994). Suppression of the humoral immune response by cannabinoids is partially mediated through inhibition of adenylate cyclase by a pertussis toxin-sensitive G-protein coupled mechanism. Biochem. Pharmacol..

[46] Schatz A.R., Kessler F.K., Kaminski N.E. (1992). Inhibition of adenylate cyclase by delta 9-tetrahydrocannabinol in mouse spleen cells: A potential mechanism for cannabinoid-mediated immunosuppression. Life Sci..

[47] Zou S., Kumar U. (2018). Cannabinoid receptors and the endocannabinoid system: Signaling and function in the central nervous system. Int. J. Mol. Sci..

[48] López-Cardona A.P., Pérez-Cerezales S., Fernández-González R., Laguna-Barraza R., Pericuesta E., Agirregoitia N., Gutiérrez-Adán A., Agirregoitia E. (2017). CB1 cannabinoid receptor drives oocyte maturation and embryo development via PI3K/Akt and MAPK pathways. FASEB J..

[49] Yan L., Luo H., Tang X., Wang H. (2023). Cannabinoids inhibit ethanol-induced activation of liver toxicity in rats through JNK/ERK/MAPK signaling pathways. J. Biochem. Mol. Toxicol..

[50] Mukhopadhyay P., Pan H., Rajesh M., Bátkai S., Patel V., Harvey-White J., Mukhopadhyay B., Haskó G., Gao B., Mackie K. (2010). CB1 cannabinoid receptors promote oxidative/nitrosative stress, inflammation and cell death in a murine nephropathy model. Br. J. Pharmacol..

[51] Drummond B.E., Ercanbrack W.S., Wingert R.A. (2023). Modeling Podocyte Ontogeny and Podocytopathies with the Zebrafish. J. Dev. Biol..

[52] Bussolati B., Deregibus M.C., Fonsato V., Doublier S., Spatola T., Procida S., Di Carlo F., Camussi G. (2005). Statins prevent oxidized LDL-induced injury of glomerular podocytes by activating the phosphatidylinositol 3-kinase/AKT-signaling pathway. J. Am. Soc. Nephrol..

[53] Bridgewater D.J., Ho J., Sauro V., Matsell D.G. (2005). Insulin-like growth factors inhibit podocyte apoptosis through the PI3 kinase pathway. Kidney Int..

[54] Jourdan T., Szanda G., Rosenberg A.Z., Tam J., Earley B.J., Godlewski G., Cinar R., Liu Z., Liu J., Ju C. (2014). Overactive cannabinoid 1 receptor in podocytes drives type 2 diabetic nephropathy. Proc. Natl. Acad. Sci. USA.

[55] de Boer I.H., Khunti K., Sadusky T., Tuttle K.R., Neumiller J.J., Rhee C.M., Rosas S.E., Rossing P., Bakris G. (2022). Diabetes management in chronic kidney disease: A consensus report by the american diabetes association (ADA) and kidney disease: Improving global outcomes (KDIGO). Diabetes Care.

[56] Rohbeck E., Eckel J., Romacho T. (2021). Cannabinoid receptors in metabolic regulation and diabetes. Physiology.

[57] Barutta F., Corbelli A., Mastrocola R., Gambino R., Di Marzo V., Pinach S., Rastaldi M.P., Perin P.C., Gruden G. (2010). Cannabinoid receptor 1 blockade ameliorates albuminuria in experimental diabetic nephropathy. Diabetes.

[58] Nam D.H., Lee M.H., Kim J.E., Song H.K., Kang Y.S., Lee J.E., Kim H.W., Cha J.J., Hyun Y.Y., Kim S.H. (2012). Blockade of cannabinoid receptor 1 improves insulin resistance, lipid metabolism, and diabetic nephropathy in db/db mice. Endocrinology.

[59] Barutta F., Bellini S., Mastrocola R., Gambino R., Piscitelli F., di Marzo V., Corbetta B., Vemuri V.K., Makriyannis A., Annaratone L. (2018). Reversal of albuminuria by combined AM6545 and perindopril therapy in experimental diabetic nephropathy. Br. J. Pharmacol..

[60] Jourdan T., Park J.K., Varga Z.V., Pálóczi J., Coffey N.J., Rosenberg A.Z., Godlewski G., Cinar R., Mackie K., Pacher P. (2018). Cannabinoid-1 receptor deletion in podocytes mitigates both glomerular and tubular dysfunction in a mouse model of diabetic nephropathy. Diabetes Obes. Metab..

[61] Raja P., Maxwell A.P., Brazil D.P. (2021). The potential of albuminuria as a biomarker of diabetic complications. Cardiovasc. Drugs Ther..

[62] Kogot-Levin A., Hinden L., Riahi Y., Israeli T., Tirosh B., Cerasi E., Mizrachi E.B., Tam J., Mosenzon O., Leibowitz G. (2020). Proximal tubule mTORC1 is a central player in the pathophysiology of diabetic nephropathy and its correction by SGLT2 inhibitors. Cell Rep..

[63] Hinden L., Ahmad M., Hamad S., Nemirovski A., Szanda G., Glasmacher S., Kogot-Levin A., Abramovitch R., Thorens B., Gertsch J. (2022). Opposite physiological and pathological mTORC1-mediated roles of the CB1 receptor in regulating renal tubular function. Nat. Commun..

[64] Jenkin K.A., McAinch A.J., Grinfeld E., Hryciw D.H. (2010). Role for cannabinoid receptors in human proximal tubular hypertrophy. Cell. Physiol. Biochem..

[65] Udi S., Hinden L., Earley B., Drori A., Reuveni N., Hadar R., Cinar R., Nemirovski A., Tam J. (2017). Proximal tubular cannabinoid-1 receptor regulates obesity-induced CKD. J. Am. Soc. Nephrol..

[66] Anders H., Huber T.B., Isermann B., Schiffer M. (2018). CKD in diabetes: Diabetic kidney disease versus nondiabetic kidney disease. Nat. Rev. Nephrol..

[67] Prakoura N., Hadchouel J., Chatziantoniou C. (2019). Novel targets for therapy of renal fibrosis. J. Histochem. Cytochem..

[68] Golosova D., Levchenko V., Kravtsova O., Palygin O., Staruschenko A. (2022). Acute and long-term effects of cannabinoids on hypertension and kidney injury. Sci. Rep..

[69] Sato M., Muragaki Y., Saika S., Roberts A.B., Ooshima A. (2003). Targeted disruption of TGF-beta1/Smad3 signaling protects against renal tubulointerstitial fibrosis induced by unilateral ureteral obstruction. J. Clin. Investig..

[70] Wu W., Wang X., Yu X., Lan H. (2022). Smad3 signatures in renal inflammation and fibrosis. Int. J. Biol. Sci..

[71] Meng X., Tang P.M., Li J., Lan H.Y. (2015). TGF-β/smad signaling in renal fibrosis. Front. Physiol..

[72] Chen D., Tang H., Jiang H., Sun L., Zhao W., Qian F. (2022). ACPA alleviates bleomycin-induced pulmonary fibrosis by inhibiting TGF-β-Smad2/3 signaling-mediated lung fibroblast activation. Front. Pharmacol..

[73] Yoshinaga T., Uwabe K., Naito S., Higashino K., Nakano T., Numata Y., Kihara A. (2016). AM251 suppresses epithelial-mesenchymal transition of renal tubular epithelial cells. PLoS ONE.

[74] Miranda K., Mehrpouya-Bahrami P., Nagarkatti P.S., Nagarkatti M. (2019). Cannabinoid receptor 1 blockade attenuates obesity and adipose tissue type 1 inflammation through miR-30e-5p regulation of delta-like-4 in macrophages and consequently downregulation of Th1 cells. Front. Immunol..

[75] Janiak P., Poirier B., Bidouard J., Cadrouvele C., Pierre F., Gouraud L., Barbosa I., Dedio J., Maffrand J.P., Le Fur G. (2007). Blockade of cannabinoid CB1 receptors improves renal function, metabolic profile, and increased survival of obese Zucker rats. Kidney Int..

[76] Wang Y., Yu Y., Zhang H., Chen C., Wan H., Chen Y., Xia F., Yu S., Wang N., Ye L. (2022). Cardiovascular and renal burdens among patients with MAFLD and NAFLD in China. Front. Endocrinol..

[77] Udi S., Hinden L., Ahmad M., Drori A., Iyer M.R., Cinar R., Herman-Edelstein M., Tam J. (2020). Dual inhibition of cannabinoid CB1 receptor and inducible NOS attenuates obesity-induced chronic kidney disease. Br. J. Pharmacol..

[78] Abuyassin B., Badran M., Ayas N.T., Laher I. (2018). Intermittent hypoxia causes histological kidney damage and increases growth factor expression in a mouse model of obstructive sleep apnea. PLoS ONE.

[79] Dou Z., Gao X., Jia Y., Chen J., Yang J., Chen Y., Wu S.J., Liu T., Wang M.T., Yang C. (2020). CB1 receptor antagonist rimonabant protects against chronic intermittent hypoxia-induced bone metabolism disorder and destruction in rats. Sleep Breath..

[80] Lee Y., Hung S., Wang H., Lin C., Wang H., Chen S., Chang M.Y., Ho L.C., Chen Y.T., Liou H.H. (2015). Sleep apnea and the risk of chronic kidney disease: A nationwide population-based cohort study. Sleep.

[81] Zhao L., Liu T., Dou Z., Wang M., Hu Z., Wang B. (2021). CB1 receptor antagonist rimonabant protects against chronic intermittent hypoxia-induced renal injury in rats. BMC Nephrol..

[82] Malek M., Nematbakhsh M. (2015). Renal ischemia/reperfusion injury; from pathophysiology to treatment. J. Renal Inj. Prev..

[83] Zhang Z., Zhao J., Dong W., Remer E., Li J., Demirjian S., Zabell J., Campbell S.C. (2016). Acute kidney injury after partial nephrectomy: Role of parenchymal mass reduction and ischemia and impact on subsequent functional recovery. Eur. Urol..

[84] Pacher P., Haskó G. (2008). Endocannabinoids and cannabinoid receptors in ischaemia-reperfusion injury and preconditioning. Br. J. Pharmacol..

[85] Sampaio L.S., Iannotti F.A., Veneziani L., Borelli-Tôrres R.T., De Maio F., Piscitelli F., Reis R.A.M., Di Marzo V., Einicker-Lamas M. (2018). Experimental ischemia/reperfusion model impairs endocannabinoid signaling and Na+/K+ ATPase expression and activity in kidney proximal tubule cells. Biochem. Pharmacol..

[86] Li X., Liu Y., Gong D., Hai K., Ke B., Zuo Y. (2020). The critical role of cannabinoid receptor 2 in URB602-induced protective effects against renal ischemia-reperfusion injury in the rat. Shock.

[87] Zhou S., Wu Q., Lin X., Ling X., Miao J., Liu X., Hu C., Zhang Y., Jia N., Hou F.F. (2021). Cannabinoid receptor type 2 promotes kidney fibrosis through orchestrating β-catenin signaling. Kidney Int..

[88] Rothner A., Gov T., Hinden L., Nemirovski A., Tam J., Rosenzweig B. (2023). Systemic changes in endocannabinoids and endocannabinoid-like molecules in response to partial nephrectomy-induced ischemia in humans. Int. J. Mol. Sci..

[89] McSweeney K.R., Gadanec L.K., Qaradakhi T., Ali B.A., Zulli A., Apostolopoulos V. (2021). Mechanisms of cisplatin-induced acute kidney injury: Pathological mechanisms, pharmacological interventions, and genetic mitigations. Cancers.

[90] Wang D., Lippard S.J. (2005). Cellular processing of platinum anticancer drugs. Nat. Rev. Drug Discov..

[91] Ries F., Klastersky J. (1986). Nephrotoxicity induced by cancer chemotherapy with special emphasis on cisplatin toxicity. Am. J. Kidney Dis..

[92] Zhang B., Ramesh G., Norbury C.C., Reeves W.B. (2007). Cisplatin-induced nephrotoxicity is mediated by tumor necrosis factor-α produced by renal parenchymal cells. Kidney Int..

[93] Pertwee R.G. (2006). Cannabinoid pharmacology: The first 66 years. Br. J. Pharmacol..

[94] Castaneto M.S., Gorelick D.A., Desrosiers N.A., Hartman R.L., Pirard S., Huestis M.A. (2014). Synthetic cannabinoids: Epidemiology, pharmacodynamics, and clinical implications. Drug Alcohol Depend..

[95] Seely K.A., Lapoint J., Moran J.H., Fattore L. (2012). Spice drugs are more than harmless herbal blends: A review of the pharmacology and toxicology of synthetic cannabinoids. Prog. Neuropsychopharmacol. Biol. Psychiatry.

[96] Wood K.E. (2013). Exposure to bath salts and synthetic tetrahydrocannabinol from 2009 to 2012 in the United States. J. Pediatr..

[97] Kamijo Y., Takai M., Fujita Y., Sakamoto T. (2016). A multicenter retrospective survey of poisoning after consumption of products containing novel psychoactive substances from 2013 to 2014 in Japan. Am. J. Drug Alcohol Abuse.

[98] Luciano R.L., Perazella M.A. (2014). Nephrotoxic effects of designer drugs: Synthetic is not better!. Nat. Rev. Nephrol..

[99] Tait R.J., Caldicott D., Mountain D., Hill S.L., Lenton S. (2016). A systematic review of adverse events arising from the use of synthetic cannabinoids and their associated treatment. Clin. Toxicol..

[100] Alp A., Akdam H., Avcıoğlu B.Y., Ersan S. (2017). Synthetic cannabinoids in the kidneys. Rev. Assoc. Med. Bras..

[101] Acharya R., Zeng X., Upadhyay K. (2023). Synthetic cannabinoid-associated acute interstitial nephritis: An emerging cause of pediatric acute kidney injury?. Clin. Nephrol. Case Stud..

[102] Kazory A., Aiyer R. (2013). Synthetic marijuana and acute kidney injury: An unforeseen association. Clin. Kidney J..

[103] D’Errico S., Zanon M., Radaelli D., Concato M., Padovano M., Scopetti M., Frati P., Fineschi V. (2022). Acute Kidney Injury (AKI) in young synthetic cannabinoids abusers. Biomedicines.

[104] Bhanushali G.K., Jain G., Fatima H., Leisch L.J., Thornley-Brown D. (2013). AKI associated with synthetic cannabinoids: A case series. Clin. J. Am. Soc. Nephrol..

[105] (2012). Acute Kidney Injury Associated with Synthetic Cannabinoid Use—Multiple States. https://www.cdc.gov/mmwr/preview/mmwrhtml/mm6206a1.htm?s_cid=mm6206a1_w.

[106] Riederer A.M., Campleman S.L., Carlson R.G., Boyer E.W., Manini A.F., Wax P.M., Brent J.A., Toxicology Investigators Consortium (ToxIC) (2016). Acute poisonings from synthetic cannabinoids—50 U.S. toxicology investigators consortium registry sites, 2010–2015. MMWR. Morb. Mort. Wkly Rep..

[107] Martins C.J., Genelhu V., Di Marzo V., Francischetti E.A. (2014). The endocannabinoid system--back to the scene of cardiometabolic risk factors control?. Horm. Metab. Res..

[108] Silvestri C., Di Marzo V. (2013). The endocannabinoid system in energy homeostasis and the etiopathology of metabolic disorders. Cell Metab..

[109] Moreira F.A., Grieb M., Lutz B. (2009). Central side-effects of therapies based on CB1 cannabinoid receptor agonists and antagonists: Focus on anxiety and depression. Best Pract. Res. Clin. Endocrinol. Metab..

[110] Topol E.J., Bousser M.G., Fox K.A., Creager M.A., Despres J.P., Easton J.D., Hamm C.W., Montalescot G., Steg P.G., Pearson T.A. (2010). Rimonabant for prevention of cardiovascular events (CRESCENDO): A randomised, multicentre, placebo-controlled trial. Lancet.

[111] Fulp A., Zhang Y., Bortoff K., Seltzman H., Snyder R., Wiethe R., Amato G., Maitra R. (2016). Pyrazole antagonists of the CB1 receptor with reduced brain penetration. Bioorg. Med. Chem..

[112] Fulp A., Bortoff K., Seltzman H., Zhang Y., Mathews J., Snyder R., Fennell T., Maitra R. (2012). Design and synthesis of cannabinoid receptor 1 antagonists for peripheral selectivity. J. Med. Chem..

[113] Cinar R., Iyer M.R., Kunos G. (2020). The therapeutic potential of second and third generation CB_1_R antagonists. Pharmacol. Ther..

[114] Chorvat R.J., Berbaum J., Seriacki K., McElroy J.F. (2012). JD-5006 and JD-5037: Peripherally restricted (PR) cannabinoid-1 receptor blockers related to SLV-319 (Ibipinabant) as metabolic disorder therapeutics devoid of CNS liabilities. Bioorg. Med. Chem. Lett..

[115] Chen W., Shui F., Liu C., Zhou X., Li W., Zheng Z., Fu W., Wang L. (2017). Novel peripherally restricted cannabinoid 1 receptor selective antagonist TXX-522 with prominent weight-loss efficacy in diet induced obese mice. Front. Pharmacol..

[116] Cluny N.L., Vemuri V.K., Chambers A.P., Limebeer C.L., Bedard H., Wood J.T., Lutz B., Zimmer A., Parker L.A., Makriyannis A. (2010). A novel peripherally restricted cannabinoid receptor antagonist, AM6545, reduces food intake and body weight, but does not cause malaise, in rodents. Br. J. Pharmacol..

[117] Eid B.G., Neamatallah T., Hanafy A., El-Bassossy H.M., Binmahfouz L., Aldawsari H.M., Hasan A., El-Aziz G.A., Vemuri K., Makriyannis A. (2021). Interference with TGFβ1-mediated inflammation and fibrosis underlies reno-protective effects of the CB1 receptor neutral antagonists AM6545 and AM4113 in a rat model of metabolic syndrome. Molecules.

[118] Barutta F., Bruno G., Mastrocola R., Bellini S., Gruden G. (2018). The role of cannabinoid signaling in acute and chronic kidney diseases. Kidney Int..

[119] Tan S., Liu H., Ke B., Jiang J., Wu B. (2020). The peripheral CB_1_ receptor antagonist JD5037 attenuates liver fibrosis via a CB_1_receptor/β-arrestin1/Akt pathway. Br. J. Pharmacol..

[120] Jourdan T., Godlewski G., Cinar R., Bertola A., Szanda G., Liu J., Tam J., Han T., Mukhopadhyay B., Skarulis M.C. (2013). Activation of the Nlrp3 inflammasome in infiltrating macrophages by endocannabinoids mediates beta cell loss in type 2 diabetes. Nat. Med..

[121] Cinar R., Iyer M.R., Liu Z., Cao Z., Jourdan T., Erdelyi K., Godlewski G., Szanda G., Liu J., Park J.K. (2016). Hybrid inhibitor of peripheral cannabinoid-1 receptors and inducible nitric oxide synthase mitigates liver fibrosis. JCI Insight.

[122] Remiszewski P., Pędzińska-Betiuk A., Mińczuk K., Schlicker E., Klimek J., Dzięcioł J., Malinowska B. (2022). Effects of the peripheral CB1 receptor antagonist JD5037 in mono– and polytherapy with the AMPK activator metformin in a monocrotaline-induced rat model of pulmonary hypertension. Front. Pharmacol..

[123] Cinar R., Iyer M.R., Kunos G. (2022). Dual inhibition of CB_1_ receptors and iNOS, as a potential novel approach to the pharmacological management of acute and long COVID-19. Br. J. Pharmacol..

[124] Mechoulam R., Parker L.A., Gallily R. (2002). Cannabidiol: An overview of some pharmacological aspects. J. Clinical Pharmacol..

[125] Pacher P., Bátkai S., Kunos G. (2006). The endocannabinoid system as an emerging target of pharmacotherapy. Pharmacol. Rev..

[126] Khoury M., Cohen I., Bar-Sela G. (2022). “The Two Sides of the Same Coin”-medical cannabis, cannabinoids and immunity: Pros and cons explained. Pharmaceutics.

[127] Almogi-Hazan O., Or R. (2020). Cannabis, the endocannabinoid system and immunity-the journey from the bedside to the bench and back. Int. J. Mol. Sci..

[128] Aziz A.I., Nguyen L.C., Oumeslakht L., Bensussan A., Ben Mkaddem S. (2023). Cannabinoids as immune system modulators: Cannabidiol potential therapeutic approaches and limitations. Cannabis Cannabinoid Res..

[129] Cunha J.M., Carlini E.A., Pereira A.E., Ramos O.L., Pimentel C., Gagliardi R., Sanvito W.L., Lander N., Mechoulam R. (1980). Chronic administration of cannabidiol to healthy volunteers and epileptic patients. Pharmacology.

[130] Mechoulam R., Peters M., Murillo-Rodriguez E., Hanus L.O. (2007). Cannabidiol–recent advances. Chem. Biodivers..

[131] Laprairie R.B., Bagher A.M., Kelly M.E.M., Denovan-Wright E.M. (2015). Cannabidiol is a negative allosteric modulator of the cannabinoid CB1 receptor. Br. J. Pharmacol..

[132] Pan H., Mukhopadhyay P., Rajesh M., Patel V., Mukhopadhyay B., Gao B., Haskó G., Pacher P. (2009). Cannabidiol attenuates cisplatin-induced nephrotoxicity by decreasing oxidative/nitrosative stress, inflammation, and cell death. J. Pharmacol. Exp. Ther..

[133] Hinden L., Kogot-Levin A., Tam J., Leibowitz G. (2022). Pathogenesis of diabesity-induced kidney disease: Role of kidney nutrient sensing. FEBS J..

[134] Mukhopadhyay P., Rajesh M., Pan H., Patel V., Mukhopadhyay B., Bátkai S., Gao B., Haskó G., Pacher P. (2010). Cannabinoid-2 receptor limits inflammation, oxidative/nitrosative stress, and cell death in nephropathy. Free Radic. Biol. Med..

[135] Barutta F., Piscitelli F., Pinach S., Bruno G., Gambino R., Rastaldi M.P., Salvidio G., Di Marzo V., Cavallo Perin P., Gruden G. (2011). Protective role of cannabinoid receptor type 2 in a mouse model of diabetic nephropathy. Diabetes.

[136] Horváth B., Mukhopadhyay P., Kechrid M., Patel V., Tanchian G., Wink D.A., Gertsch J., Pacher P. (2012). β-Caryophyllene ameliorates cisplatin-induced nephrotoxicity in a cannabinoid 2 receptor-dependent manner. Free Radic. Biol. Med..

[137] Barutta F., Grimaldi S., Franco I., Bellini S., Gambino R., Pinach S., Corbelli A., Bruno G., Rastaldi M.P., Aveta T. (2014). Deficiency of cannabinoid receptor of type 2 worsens renal functional and structural abnormalities in streptozotocin-induced diabetic mice. Kidney Int..

[138] Mukhopadhyay P., Baggelaar M., Erdelyi K., Cao Z., Cinar R., Fezza F., Ignatowska-Janlowska B., Wilkerson J., van Gils N., Hansen T. (2016). The novel, orally available and peripherally restricted selective cannabinoid CB2 receptor agonist LEI-101 prevents cisplatin-induced nephrotoxicity. Br. J. Pharmacol..

[139] Pressly J.D., Mustafa S.M., Adibi A.H., Alghamdi S., Pandey P., Roy K.K., Doerksen R.J., Moore B.M., Park F. (2018). Selective cannabinoid 2 receptor stimulation reduces tubular epithelial cell damage after renal ischemia-reperfusion injury. J. Pharmacol. Exp. Ther..

[140] Pressly J.D., Soni H., Jiang S., Wei J., Liu R., Moore B.M., Adebiyi A., Park F. (2019). Activation of the cannabinoid receptor 2 increases renal perfusion. Physiol. Genom..

[141] Trojnar E., Erdelyi K., Matyas C., Zhao S., Paloczi J., Mukhopadhyay P., Varga Z.V., Hasko G., Pacher P. (2020). Cannabinoid-2 receptor activation ameliorates hepatorenal syndrome. Free Radic. Biol. Med..

[142] Swanson M.L., Regner K.R., Moore B.M., Park F. (2022). Cannabinoid type 2 receptor activation reduces the progression of kidney fibrosis using a mouse model of unilateral ureteral obstruction. Cannabis Cannabinoid Res..

